# The Lipid-Binding Defective Dynamin 2 Mutant in Charcot-Marie-Tooth Disease Impairs Proper Actin Bundling and Actin Organization in Glomerular Podocytes

**DOI:** 10.3389/fcell.2022.884509

**Published:** 2022-05-10

**Authors:** Eriko Hamasaki, Natsuki Wakita, Hiroki Yasuoka, Hikaru Nagaoka, Masayuki Morita, Eizo Takashima, Takayuki Uchihashi, Tetsuya Takeda, Tadashi Abe, Ji-Won Lee, Tadahiro Iimura, Moin A Saleem, Naohisa Ogo, Akira Asai, Akihiro Narita, Kohji Takei, Hiroshi Yamada

**Affiliations:** ^1^ Department of Neuroscience, Graduate School of Medicine, Dentistry and Pharmaceutical Sciences, Okayama University, Okayama, Japan; ^2^ Division of Malaria Research, Proteo-Science Center, Ehime University, Matsuyama, Japan; ^3^ Department of Physics, Nagoya University, Nagoya, Japan; ^4^ Department of Pharmacology, Faculty and Graduate School of Dental Medicine, Hokkaido University, Sapporo, Japan; ^5^ Bristol Renal, Translational Health Sciences, Bristol Medical School, University of Bristol, Bristol, United Kingdom; ^6^ Center for Drug Discovery, Graduate School of Pharmaceutical Sciences, University of Shizuoka, Shizuoka, Japan; ^7^ Graduate School of Science, Nagoya University, Nagoya, Japan

**Keywords:** dynamin, podocyte, actin, bundle, GTPase, CMT

## Abstract

Dynamin is an endocytic protein that functions in vesicle formation by scission of invaginated membranes. Dynamin maintains the structure of foot processes in glomerular podocytes by directly and indirectly interacting with actin filaments. However, molecular mechanisms underlying dynamin-mediated actin regulation are largely unknown. Here, biochemical and cell biological experiments were conducted to uncover how dynamin modulates interactions between membranes and actin in human podocytes. Actin-bundling, membrane tubulating, and GTPase activities of dynamin were examined *in vitro* using recombinant dynamin 2-wild-type (WT) or dynamin 2-K562E, which is a mutant found in Charcot-Marie-Tooth patients. Dynamin 2-WT and dynamin 2-K562E led to the formation of prominent actin bundles with constant diameters. Whereas liposomes incubated with dynamin 2-WT resulted in tubule formation, dynamin 2-K562E reduced tubulation. Actin filaments and liposomes stimulated dynamin 2-WT GTPase activity by 6- and 20-fold, respectively. Actin-filaments, but not liposomes, stimulated dynamin 2-K562E GTPase activity by 4-fold. Self-assembly-dependent GTPase activity of dynamin 2-K562E was reduced to one-third compared to that of dynamin 2-WT. Incubation of liposomes and actin with dynamin 2-WT led to the formation of thick actin bundles, which often bound to liposomes. The interaction between lipid membranes and actin bundles by dynamin 2-K562E was lower than that by dynamin 2-WT. Dynamin 2-WT partially colocalized with stress fibers and actin bundles based on double immunofluorescence of human podocytes. Dynamin 2-K562E expression resulted in decreased stress fiber density and the formation of aberrant actin clusters. Dynamin 2-K562E colocalized with α-actinin-4 in aberrant actin clusters. Reformation of stress fibers after cytochalasin D-induced actin depolymerization and washout was less effective in dynamin 2-K562E-expressing cells than that in dynamin 2-WT. Bis-T-23, a dynamin self-assembly enhancer, was unable to rescue the decreased focal adhesion numbers and reduced stress fiber density induced by dynamin 2-K562E expression. These results suggest that the low affinity of the K562E mutant for lipid membranes, and atypical self-assembling properties, lead to actin disorganization in HPCs. Moreover, lipid-binding and self-assembly of dynamin 2 along actin filaments are required for podocyte morphology and functions. Finally, dynamin 2-mediated interactions between actin and membranes are critical for actin bundle formation in HPCs.

## Introduction

Glomerular podocytes are highly differentiated epithelial cells that line the urinary side of the glomerular basement membrane. They participate in filtration of blood plasma to form primary urine. Podocytes have a complex architecture. They consist of major primary processes that branch to form secondary and tertiary foot processes. These secondary and tertiary branches interdigitate with those of neighboring podocytes to form and maintain the glomerular slit diaphragms (Pavenstädt et al., 2003). Foot processes of podocytes are mainly supported by the actin cytoskeleton, including actin bundles and cortical actin mesh structures (Sever and Schiffer, 2018). Proper regulation of the actin cytoskeleton is crucial for maintaining the unique morphology and the filtering function of podocytes.

**FIGURE 1:**
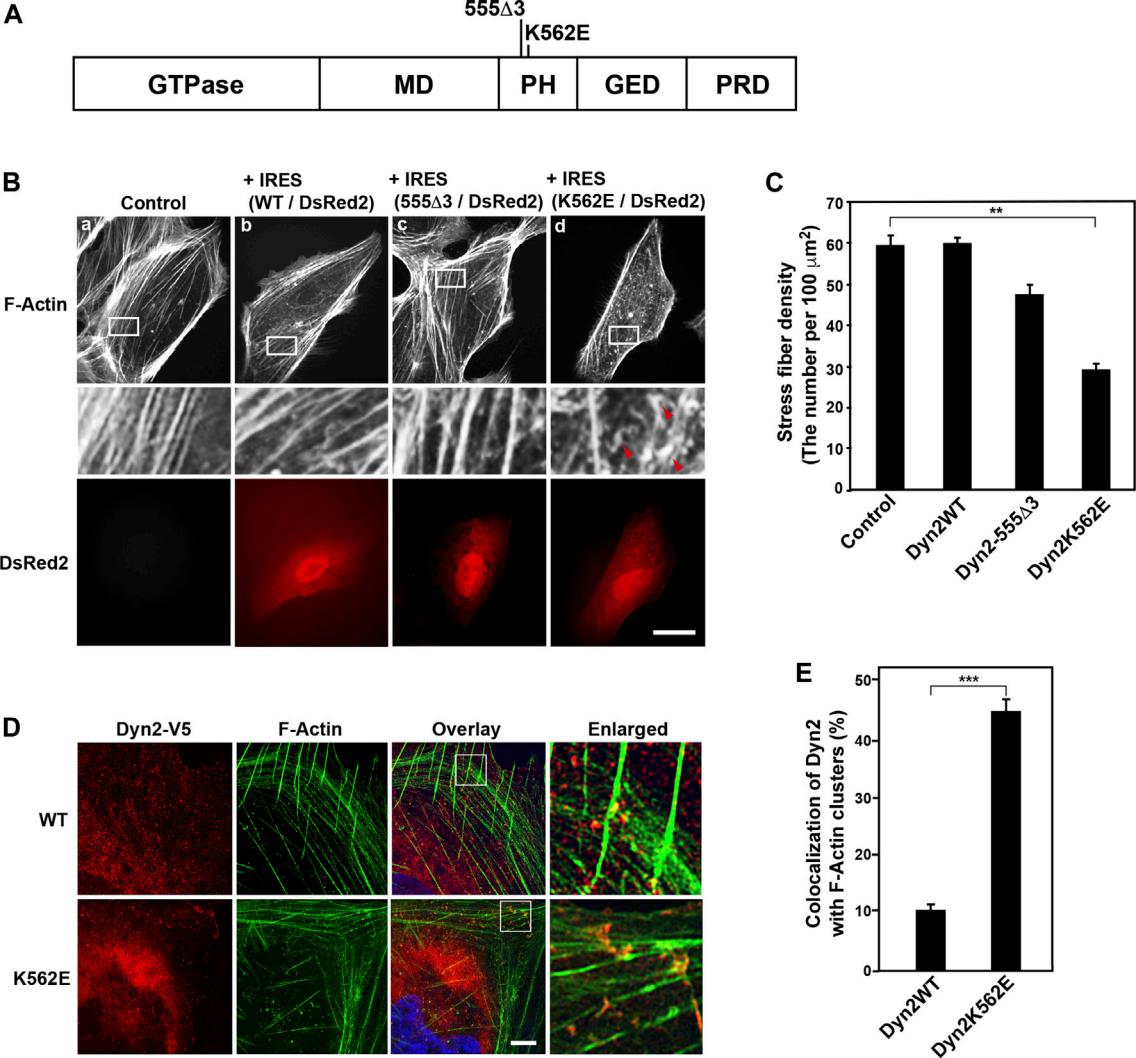
Expression of the dynamin 2 (Dyn2)-K562E mutant leads to the formation of aberrant actin bundles and a decrease in stress fiber density in human podocyte cells. **(A)** Domain structure of dynamin 2 and the location of the 555Δ3 and K562E mutations in rat dynamin 2. GTPase domain, MD = Middle Domain; PH = Pleckstrin Homology domain; GED = GTPase Effector Domain; PRD = Proline arginin-Rich Domain. **(B)** Alexa Fluor 488-phalloidin-labeled actin filaments in HPCs. HPCs were transfected with dynamin 2-WT (WT) **(b)**, 555Δ3 **(c)**, or K562E **(d)** expression constructs cloned into the pIRES-DsRed2 vector. Non-transfected cells (Controls) are shown in **(a)**. White rectangular areas in the top panels correspond to enlarged images in the middle panels. Stress fibers are clearly observed in control and dynamin 2-WT-expressing cells. Note the reduction in actin bundles and stress fibers, and the presence of F-Actin clusters (red arrowheads) in K562E mutant-expressing cells **(d)**. Dynamin-transfected cells were identified by DsRed2 expression (bottom panels). Actin clusters were shown by arrowheads. Scale bars: 20 μm in top and bottom panels and 3.6 μm in middle panels. **(C)** Stress fiber density in control HPCs and those expressing the various dynamin 2 constructs. Data are means ± S.E.M. of 33 cells (control), 101 cells (dynamin 2-WT), 39 cells (dynamin 2-555Δ3) or 105 cells (dynamin 2-K562E) from three independent experiments. (**p < 0.01). **(D)** Super-resolution microscopy images of V5-tagged dynamin 2-WT or K562E, and actin filaments (F-Actin) in HPCs processed by double-immunofluorescence. White squares in the overlay images correspond to the enlarged images on the right. Scale bars: 5 μm, 1 μm in enlarged panels. **(E)** Quantification of the colocalization of dynamin 2-WT or K562E, and actin filaments in HPCs. Data are mean ± S.E.M. of 51 cells in dynamin 2-WT and 50 cells in K562E from three independent experiments. For each sample, colocalization was determined in three randomly selected areas per cell (21 μm^2^). (***p < 0.001).

Dynamins are endocytic proteins that form vesicles by fission of invaginated plasma membranes (Antonny et al., 2016). Dynamins are divided into three isoforms in mammals (Ferguson and De Camilli, 2012). Dynamin 1 is enriched in the brain, dynamin 2 is expressed in all cells, and dynamin 3 localizes to the brain, lung, and testis (Nakata et al., 1993). All dynamin isoforms contain an N-terminal GTPase domain, a middle domain (MD), phosphoinositide-binding pleckstrin homology (PH) domain, GTPase effector domain (GED), and C-terminal proline and arginine-rich domain (PRD) which interacts with variety of proteins containing the Src-homology-3 domain (Figure 1A; Ferguson and De Camilli, 2012). Crystallographically, the three α-helix of MD and the single α-helix in GED form four-helix bundle structure termed the “stalk”, which provides interface required for dimerization and self-assembly (Faelber et al., 2011; Ford et al., 2011). In unassembled dynamin, PH domain is at “closed” position, at which it is folded back onto the stalk preventing oligomerization of dynamin. Upon binding of dynamin to phosphatidylinositol (4,5) bisphosphate (PIP_2_) -containing membrane, the PH domain is displaced to “open” dynamin conformation capable of self-assembly (Srinivasan et al., 2016; Kong et al., 2018). Mutations in dynamin 2 gene cause autosomal dominantly inherited diseases, Charcot-Marie-Tooth disease (CMT) (Züchner et al., 2005), and centronuclear myopathy (CNM) (Bitoun et al., 2005), which are characterized by degenerative changes of peripheral nerves and muscles respectively. Although both CMT mutations and CNM mutations are mostly found in the PH domain, the two group of mutations are differentially located. While CNM mutations are present in PH domain-stalk interface, CMT mutations, including K562E used in this study, are located at the opposite side of PH domain implicated in membrane binding (Faelber et al., 2011; Tassin et al., 2021).

All dynamins function in endocytosis by participating in membrane fission (Antonny et al., 2016), and recognition of phosphoinositides by dynamin’s PH domain, especially, electrically polarised seven-stranded β-sandwich, is crucial for this function (Vallis et al., 1999). Dynamins are also involved in the regulation of the cytoskeleton. Dynamin directly or indirectly interacts with actin (Sever et al., 2013), and thus regulates actin dynamics in lamellipodia and dorsal membrane ruffles (Cao et al., 1998; McNiven et al., 2000), invadopodia (Baldassarre et al., 2003), podosomes (Ochoa et al., 2000), growth cones (Torre et al., 1994; Kurklinsky et al., 2011; Yamada et al., 2013), phagocytic cups (Gold et al., 1999; Otsuka et al., 2009), and filopodia of cancer cells (Yamada et al., 2016a). It is recently shown that dynamin helix bundles 12-16 actin filaments, and the dynamin-enriched actin bundle was present at the fusogenic synapse (Zhang et al., 2020).

Dynamin maintains the integrity and structure of the glomerular filtration barrier by regulating actin and microtubule cytoskeleton in addition to endocytosis. Podocyte-specific double knockouts of dynamin 1 and dynamin 2 in mice result in severe proteinuria and renal failure, which are caused by the disruption of glomerular slit diaphragms (Soda et al., 2012). In addition, a reduction in cellular dynamin levels by cathepsin L expression causes proteinuria in mice (Sever et al., 2007). Dynamin has been implicated in the turnover of nephrin on the surface of podocyte foot processes via endocytosis (Soda et al., 2012). Dynamin 1 in podocyte is involved in cellular protrusion formation by regulating microtubule organization and stabilization (La et al., 2020). Furthermore, dynamin participates in the maintenance of the structure of foot processes through direct and indirect interactions with actin filaments (Gu et al., 2010). Bis-T-23 was originally reported as a potent dynamin inhibitor of lipid-stimulated GTPase activity (Hill et al., 2005). Subsequently, Gu et al. found that Bis-T-23 promotes dynamin’s oligomerization *in vitro* (Gu et al., 2017), and increases the formation of stress fibers and focal adhesions in mouse podocyte cells (Gu et al., 2014). Because of these effects of Bis-T-23 on dynamin and dynamin-regulated actin-rich structures, Bis-T-23 is shown to improve proteinuria in several animal models (Schiffer et al., 2015). However, a mode of action for actin regulation by dynamin 2 remains to be elucidated.

Our previous studies show that the dynamin 2 CMT mutant K562E decreases the formation of stress fibers and triggers the assembly of aberrant actin clusters in U2OS cells (Yamada et al., 2016b). In the present study, biochemical and cell biological experiments were used to uncover the mode of action for actin regulation by dynamin 2 by comparing the effects of dynamin 2-WT on actin filaments to those of K562E in conditionally immortalized human podocytes (HPCs). Expression of dynamin 2-K562E resulted in decreased formation of stress fibers and the appearance of aberrant actin clusters. Bis-T-23 was less effective on the self-assembly of dynamin 2-K562E *in vitro*, it was unable to reverse the aberrant actin structures in dynamin 2-K562E expressing cells. Furthermore, *in vitro* experiments revealed defects in the interaction of actin bundles formed by dynamin 2-K562E to lipid membranes. These results suggest that the self-assembly and membrane interaction properties of dynamin 2 are crucial for actin regulation, which is a prerequisite for maintaining the unique morphology and filtering function of podocytes.

## Experimental Methods

### Antibodies and Reagents

Rabbit polyclonal anti-V5 antibody (AB3792), rabbit polyclonal anti-α-actinin-4 antibody (0042-05), and mouse monoclonal antibody against paxillin (AHO0492) were purchased from Sigma-Aldrich, immunoGlobe, and Thermo Fisher Scientific, respectively. Alexa Fluor 488-conjugated goat anti-mouse IgG (A11001), Rhodamine Red X-conjugated goat anti-rabbit IgG (R6394), and Alexa Fluor 488-phalloidin (A12379) were purchased from Life Technologies. The rabbit polyclonal antibodies against mouse IgG (31450) and goat IgG (31402), and goat polyclonal antibody against rabbit IgG (31460) were purchased from Thermo Fisher Scientific. The mouse monoclonal antibodies against beta-actin (A5441) and cytochalasin D (C8273) were purchased from Sigma-Aldrich. Bis-T-23 (2-cyano-N-{3-[2-cyano-3-(3,4,5-trihydroxyphenyl) acryloylamino] propyl}-3-(3,4,5 trihydroxyphenyl) acrylamide) (ab146050) was purchased from Abcam, and stored frozen as a 50 mM stock solution in dimethyl sulfoxide (DMSO).

### Cell Culture

The conditionally immortalized HPC line was cultured as described previously (Saleem et al., 2002). Briefly, the cells were cultured on type I collagen-coated plastic dishes (356450, Corning Inc.) in Roswell Park Memorial Institute (RPMI) 1640 medium (189-02025, Fujifilm Wako Pure Chemicals Co. Ltd.) containing 10% fetal bovine serum (26140079, Thermo Fisher Scientific), 100 U/ml penicillin, 100 μg/ml streptomycin (15140122, Thermo Fisher Scientific), and ITS-G (Insulin-Transferrin-Selenium mixture) (100×, 41400045, Thermo Fisher Scientific), and maintained at 33°C and 5% CO_2_. To induce differentiation, podocytes were cultured at 37°C in medium lacking ITS-G for 7–14 days. Under these conditions, the cells stopped proliferating and were positive for synaptopodin.

### Purification of Recombinant Proteins

His-tagged dynamin 2b was expressed using the Bac-to-Bac baculovirus expression system (Thermo Fisher Scientific) and purified as described previously (Yamada et al., 2016a). The purified dynamin proteins were concentrated using Centriplus YM50 (4310, Merck-Millipore). His-tagged rat dynamin 2-WT or dynamin 2-K562E was expressed using a wheat germ cell-free expression system (CellFree Sciences). Dynamin 2-WT or dynamin 2-K562E was resolved in 100 mM NaCl, 50 mM Tris, 500 mM imidazole, pH8.0, and stored at 4°C until use. We used dynamin 2-WT and K562E produced by a wheat germ cell free expression system except Supplementary Figures S2E, F, and baculovirus protein expression system was used for Supplementary Figures S2E, F.

### Liposome Preparation

Liposomes were prepared as previously described (Takeda et al., 2018). 10% (mol/mol) PIP_2_ (Cat. No 524644, Calbiochem), 80% phosphatidylethanolamine (PE; 840022C, Avanti Polar Lipids), 10% cholesterol (700000, Avanti Polar Lipids) were mixed in chloroform. Then chloroform was evaporated using slow-flow nitrogen gas to produce lipid film on the glass and then completely dried under vacuum for at least 1 day. The dried lipid was rehydrated by water-saturated nitrogen gas followed by addition of 500 μl of filtered 100 mM KCl, 10 mM Tris-HCl, pH 7.5 for 2 h at 37°C. The resultant membrane vesicles were passed through 0.4 μm- or 1 μm-polycarbonate filters respectively 11 times using Avanti Mini extruder. To visualize liposomes under fluorescent microscope, 1% Rhodamine-labeled PE was added to liposomes.

### Complementary DNA Constructs and Transfection

Rat dynamin 2-WT, 555Δ3, and K562E cloned into pcDNA4 V5/His are described in Yamada et al. (2016b). Rat dynamin 2-WT was subcloned into pIRES2-DsRed2 as an EcoRI-SmaI fragment (Clontech Laboratories). Mutations were introduced with QuikChange II XL (Agilent Technologies, Santa Clara, CA) following the manufacturer’s instructions, and mutation accuracy was verified by DNA sequencing. Vectors containing dynamin 2-WT and the site-directed mutants were transfected into cells using Lipofectamine LTX reagent (Thermo Fisher Scientific) according to the manufacturer’s protocol. After transfection for 2 days, the cells were processed by double immunofluorescence.

### Fluorescence Microscopy

HPCs were fixed in 4% paraformaldehyde and processed for immunofluorescence as described previously (Yamada et al., 2016a). For cytochalasin D washout experiments, HPCs were incubated in 5 μM cytochalasin D at 37°C for 30 min. Cytochalasin D-containing medium was then replaced with fresh cytochalasin D-free medium. Cells were further incubated for 60 min after cytochalasin D washout, and then fixed with 4% paraformaldehyde in phosphate buffered saline (PBS; 145 mM NaCl, 10 mM phosphate buffer, pH7.4).

For Bis-T-23 treatment, HPCs were incubated in 50 μM Bis-T-23 at 37°C for 30 min, and then fixed with 4% paraformaldehyde in PBS. After fixation, cells were subjected to double-immunofluorescence. Samples were examined with a spinning-disc confocal microscope system (X-Light Confocal Imager; CREST OPTICS S.P.A., Rome, Italy), which consisted of an inverted microscope (IX-71; Olympus Optical Co., Ltd., Tokyo, Japan) and an iXon + camera (Oxford Instruments, Oxfordshire, United Kingdom). The confocal system was controlled by the MetaMorph software (Molecular Devices, Sunnyvale, CA, United States). When necessary, images were processed with Adobe Photoshop CS3 or Illustrator CS3 software. The N-SIM system (NIKON Corp., Tokyo, Japan) was used for super-resolution microscopy.

### Determination of Phosphate Concentration

Dynamin 2-WT or K562E (1.5 μM) was incubated with actin filaments (1 μM) in buffer (100 mM KCl, 2 mM MgCl_2_, 0.2 mM CaCl_2_, 10 mM Tris-HCl, pH 7.5, 0.62 mM ATP, 0.5 mM DTT) at 4°C for 16 h. The dynamin 2-actin filament mixture (160 μl) was then incubated in the presence of 1 mM GTP at 37°C for 15 min. GTP hydrolysis was measured using a colorimetric assay to detect inorganic phosphate (Pi) release as previously described (Leonard et al., 2005).

### Quantification of Actin Bundles by a Low-Speed Sedimentation Assay

Non-muscle actin (APHL99, Cytoskeleton Inc.) was polymerized in F-buffer (50 mM KCl, 2 mM MgCl_2_, 0.2 mM CaCl_2_, 10 mM Tris-HCl, pH7.5, 0.5 mM DTT, 1 mM ATP) for 1 h. Dynamin 2-WT or K562E (1 μM) was then incubated with actin filaments (3 μM) in F-buffer for 1 h. Actin bundles were sedimented by low-speed centrifugation at 14,000 × g for 1 h. The pellet and supernatant were separated by SDS-PAGE, stained with SYPRO Orange (S6650, Thermo Fisher Scientific), and quantified by densitometry using Image J. All steps were carried out at room temperature.

### Transmission Electron Microscopy

Negative staining was performed according to Yamada et al. (Yamada et al., 2013). The dynamin 2-WT- or K562E-induced actin-bundling assays were conducted by incubating 1.5 μM of the dynamins with 1 μM actin filaments in F-buffer containing 50 mM or 100 mM KCl at 4°C for 16 h. The samples were absorbed onto a Formvar- and carbon-coated copper grid. To observe the effect of GTP hydrolysis on actin bundle morphology, GTP or GMP-PNP at the indicated concentrations were added onto the grid and incubated for 1 min or 5 min, respectively. The grids were then stained with 3% uranyl acetate in double deionized H_2_O for 2 min. Grids were imaged with a transmission electron microscope (TEM) (H-7650, Hitachi High-Tech Corp., Tokyo, Japan) at a voltage of 120 kV.

### Atomic Force Microscopy (AFM)

The laboratory-built AFM observation was performed as follows (Takeda et al., 2018). First, after incubation with a mixture of dynamin 2-WT/K562E and actin filament in F-buffer containing 50 mM KCl, a glass stage with a diameter of 2 mm was immersed in the solution, and the complex is fixed to the glass substrate by centrifugation (6,000 × g for 20 min). After that, the samples were washed by the F-buffer and fixed with the F-buffer with 0.01% glutaraldehyde solution. Then AFM images were obtained in the F-buffer at room temperature.

### Morphometry

Cells were stained with Alexa Fluor 488-phalloidin, and analyzed by fluorescent confocal microscopy to determine stress fiber density. The number of stress fibers in three randomly selected areas (10 μm × 10 μm) per cell was counted. Data were analyzed from three independent experiments. The extent of colocalization of dynamin 2-WT or K562E with F-actin or α-actinin-4 was assessed by imaging immunostained cells, and measuring the immunoreactivities in three randomly selected areas (5 × 5 μm) per cell. To quantify focal adhesions, cells immunostained with dynamin 2-WT or K562E, and paxillin, were imaged. The number of clear dots per cell was counted. we performed morphological analysis again. For quantification of liposomes bound to actin filaments, images of the mixture, including dynamin 2-WT or K562E, and F-actin with rhodamine-labelled liposomes, were taken with a confocal microscope at a 1000× magnification. The corresponding pixels to liposomes colocalizing at F-actin were extracted using Metamorph software from double-fluorescent overlay image (1024 × 1024 pixel) of liposome (red) and actin filament (green) and then calculated the total amount of colocalizing liposomes with F-actin. We normalized the total amount of colocalizing liposomes with F-actin by the same amount of F-actin in each condition. We quantified the pitch in dynamin-induced actin bundles by visually scanning negatively-stained samples at the electron microscope according to Takei et al. (Takei et al., 1999).

### Statistical Analysis

Data were analyzed for statistical significance using KaleidaGraph software for Macintosh, version 4.1 (Synergy Software Inc., Essex Junction, VT, United States). Student’s t-tests were used to analyze statistical significance between two groups. An analysis of variance and Tukey’s honest significant difference post-hoc test were used to compare several groups. All data are displayed as means ± standard error of the means (S.E.M.) with *p* < 0.05 considered statistically significant.

## Results

### Expression of Dynamin 2-K562E Results in Aberrant Actin Stress Fibers and Actin Clusters in Human Podocyte Cells

Our previous studies show that dynamin 2-K562E, a point mutation in dynamin 2 PH domain, induces aberrant actin filament structures in U2OS cells (Yamada et al., 2016b). To investigate the mode of action for dynamin 2 mutation-triggered changes in actin morphology in HPCs, we first examined actin filament distribution in dynamin 2-WT- or K562E-expressing cells by double-immunofluorescence microscopy. Dynamin 2-WT and K562E were compared to dynamin 2-555Δ3, which is a deletion mutant that affects microtubule organization (Figures 1A, Bc; Tanabe and Takei 2009). DsRed2 was co-expressed with the dynamin 2-WT and mutant constructs to identify cells expressing the exogenous proteins. Control HPCs, untransfected, formed the typical parallel arrangement of stress fibers and actin bundles (Figure 1Ba). The organization of the actin cytoskeleton in cells expressing dynamin 2-WT was similar to that of control cells (Figure 1Bb). Furthermore, actin filament distribution in 555Δ3-expressing cells was similar to that of control or dynamin 2-WT-expressing cells (Figures 1Bc, C). By contrast, stress fiber density was approximately 50% lower in K562E-expressing cells than that of control or dynamin 2-WT-expressing cells (Figures 1Bd, C). Dynamin 2-WT was observed as fine dots distributed throughout the cell when imaged by super-resolution microscopy. The dot-like dynamin 2-WT structures partially colocalized with stress fibers and actin bundles (Figures 1D upper panels, E). By contrast, K562E-expressing cells showed numerous actin filament clusters, and aberrant stress fibers accumulating with K562E-dynamin 2 (Figures 1D bottom panels, E). The results indicate that K562E-dynamin 2 causes distinct effects on the organization of the actin cytoskeleton in HPCs.

### Dynamin 2 is Involved in Stress Fiber Formation in Human Podocytes

**FIGURE 2:**
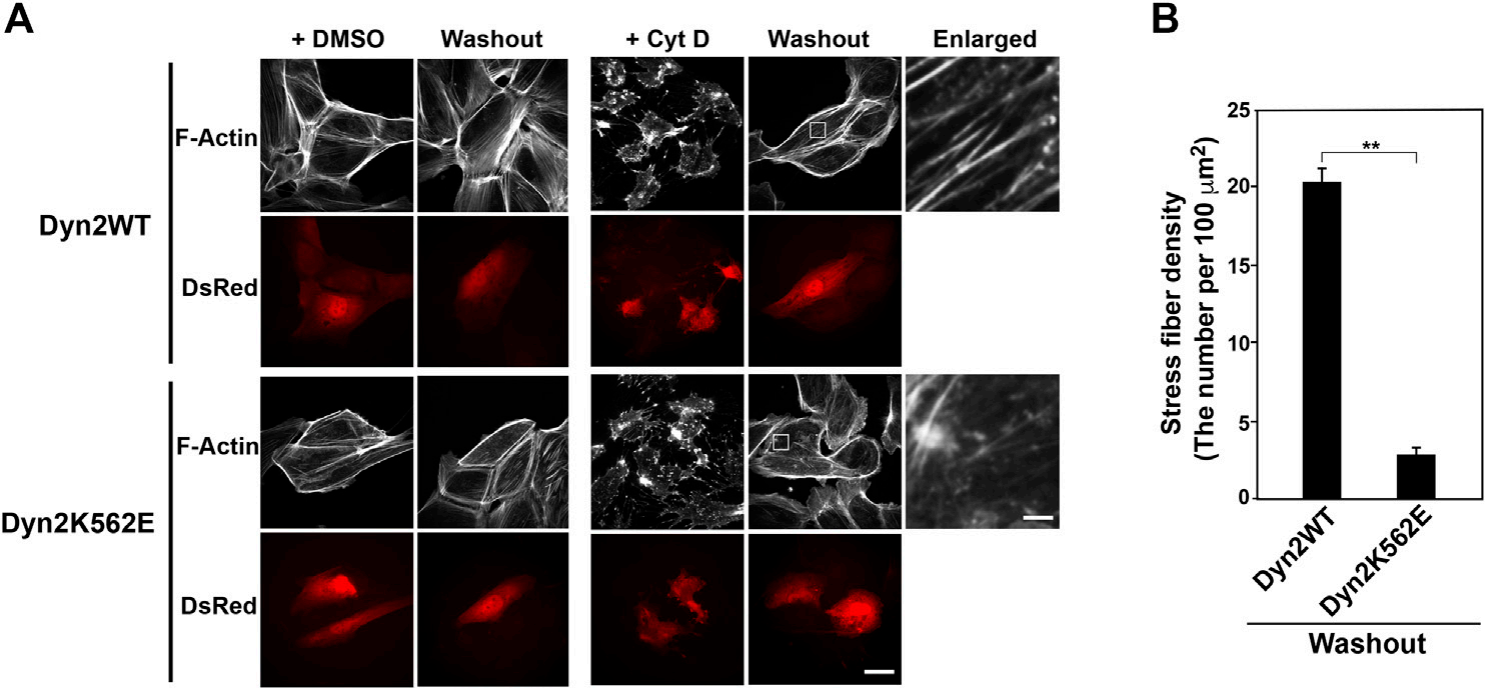
Stress fiber formation decreases in dynamin 2 (Dyn2)-K562E-expressing HPCs. **(A)** Double-immunofluorescence images of HPCs transfected with dynamin 2-WT or K562E cloned into the pIRES-DsRed2 vector as in Figure 1B. HPCs were treated with or without 5 μM cytochalasin D (Cyt D) for 30 min. Washout experiments involved changing the Cyt D-containing medium with Cyt D-free medium. After 60 min, actin filaments were stained with Alexa Fluor 488-phalloidin (Washout). Cells were treated with the solvent control (DMSO) solution represent the negative controls. Scale bars: 20 and 1.8 μm in enlarged panels. **(B)** Stress fiber density in dynamin 2-WT- or K562E-expressing HPCs after washing out of Cyt D. Data are means ± S.E.M. of 24 cells (dynamin 2-WT) or 27 cells (dynamin 2-K562E) from three independent experiments. (**p < 0.01).

Given the appearance of aberrant stress fibers and actin clusters in dynamin 2-K562E-expressing HPCs, we next asked whether the dynamin mutant affects stress fiber formation. Actin filaments, including stress fibers, are disrupted by the actin polymerization inhibitor cytochalasin D (Brenner and Korn, 1979). Reformation of stress fibers after removing cytochalasin D from the medium was examined. As shown in Figure 2A, cytochalasin D treatment for 30 min resulted in the disruption of stress fibers and actin bundles in dynamin 2-WT- and dynamin 2-K562E-expressing HPCs. Dynamin 2-WT-expressing cells reformed clear stress fibers 1 hour after removal of cytochalasin D. On the other hand, stress fiber reformation was lower in dynamin 2-K562E-expressing cells than that in dynamin 2-WT-expressing cells (Figure 2B). Thus, dynamin 2 is required in the early stages of stress fiber reformation.

**FIGURE 3:**
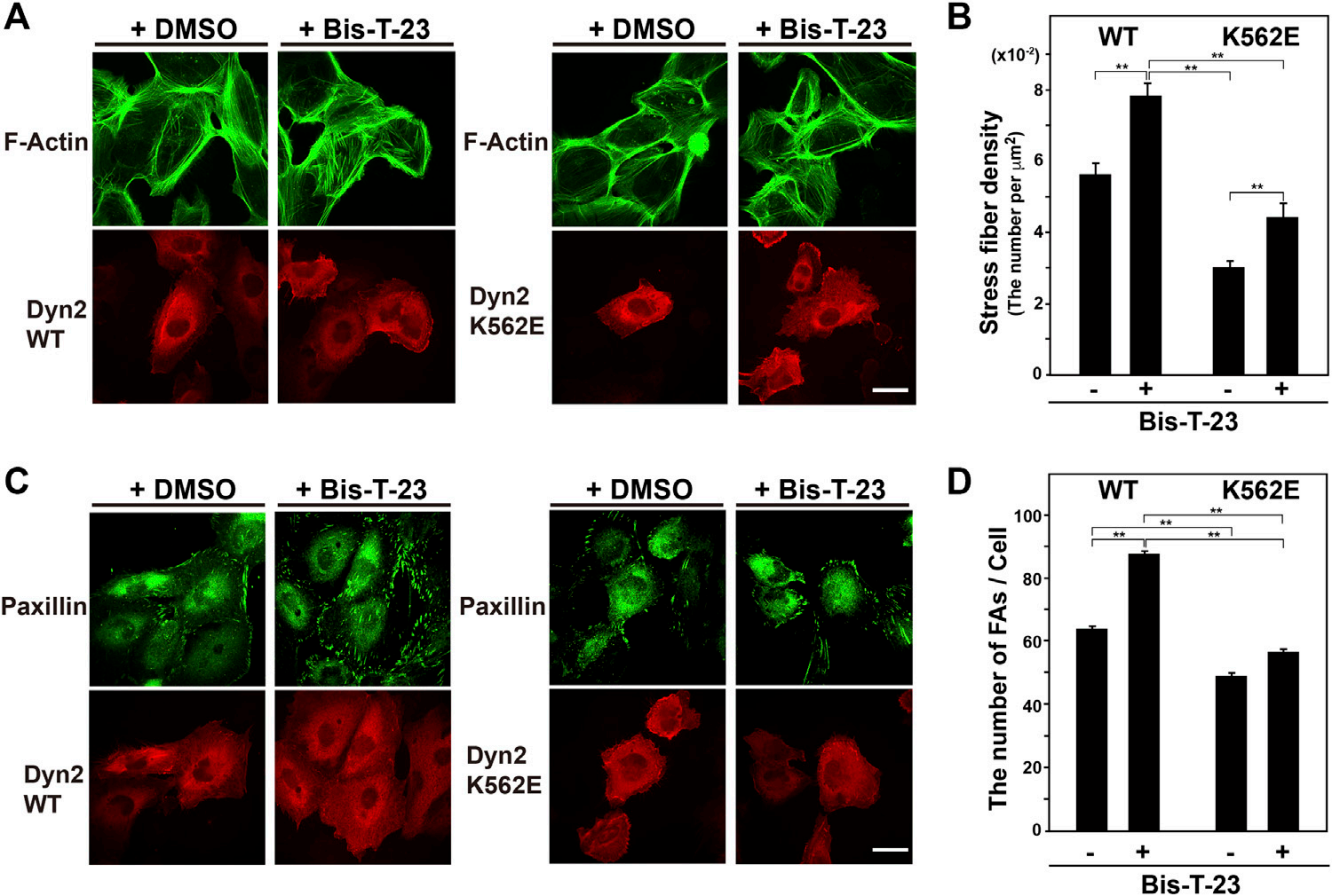
Stress fibers and focal adhesions increase in Bis-T-23-treated HPCs. **(A)** Double-immunofluorescence of V5-tagged dynamin 2 (Dyn2)-WT or K562E (bottom panels) and actin filaments (top panels) in Bis-T-23-treated (50 μM) HPCs and corresponding solvent controls (DMSO). Scale bar: 20 μm. **(B)** Stress fiber density in Bis-T-23-treated dynamin 2-WT- or K562E-expressing HPCs. Data are means ± S.E.M. of 35 cells (dynamin 2-WT/DMSO), 33 cells (dynamin 2-WT/Bis-T-23), 38 cells (dynamin 2-K562E/DMSO) or 35 cells (dynamin 2-K562E/Bis-T-23) from three independent experiments. (**p < 0.01). **(C)** Double-immunofluorescence of V5-tagged dynamin 2-WT or K562E (bottom panels) and paxillin (top panels) in Bis-T-23-treated (50 μM) HPCs and corresponding solvent controls (DMSO). Scale bar; 20 μm. **(D)** Number of focal adhesions in Bis-T-23-treated dynamin 2-WT- or K562E-expressing HPCs. Data are means ± S.E.M. of 35 cells (dynamin 2-WT/DMSO), 32 cells (dynamin 2-WT/Bis-T-23), 35 cells (dynamin 2-K562E/DMSO) or 34 cells (dynamin 2-K562E/Bis-T-23) from three independent experiments. (**p < 0.01). more than 32 cells from three independent experiments. (**p < 0.01).

The dynamin polymerization enhancer, Bis-T-23, promotes the formation of stress fibers and focal adhesions in mouse podocyte cells (Schiffer et al., 2015; Gu et al., 2017). Here, Bis-T-23 enhanced stress fiber formation in dynamin 2-K562E-expressing and dynamin 2-WT-expressing HPCs (Figures 3A,B). However, Bis-T-23-induced stress fiber formation in dynamin 2-K562E-expressing HPCs was lower than that in dynamin 2-WT-expressing HPCs. Bis-T-23 had the same effect on focal adhesion formation in dynamin 2-K562E-expressing and dynamin 2-WT-expressing HPCs (Figures 3C,D).

### The Dynamin 2 Ring-Like Structure Directly Bundles Actin Filaments

To determine if dynamin 2 directly affects actin filaments, we examined *in vitro* the GTPase activities and membrane deformation activities of recombinant dynamin 2-WT and dynamin 2-K562E. Purified dynamin 2-WT showed high GTPase activity under low ionic strength buffer (Supplementary Figure S2A) or in the presence of lipid membranes (Supplementary Figure S2B). These findings were consistent with previous reports (Warnock et al., 1997; Leonard et al., 2005). By contrast, GTPase activity of dynamin 2-K562E was approximately one-third that of dynamin 2-WT under low ionic strength buffer (Supplementary Figure S2A). Furthermore, liposome-stimulated increase of GTPase activity was not observed in dynamin 2-K562E (Supplementary Figure S2B).

For membrane deformation activity assays, liposomes containing PIP_2_ were incubated with dynamin 2-WT or dynamin 2-K562E, and examined by TEM. Dynamin 2-WT formed typical membrane tubules decorated with dynamin spiral polymers as reported previously (Chin et al., 2015; Supplementary Figure S2C). On the other hand, dynamin 2-K562E hardly tubulated the liposomes, although it slightly deformed spherical liposomes (Supplementary Figure S2C). In absence of liposomes, both dynamin 2-WT, dynamin 2-K562E formed ring-shaped polymers under GDP-Pi conditions (Supplementary Figure S2D; Carr and Hinshaw, 1997), which suggested that the ability of dynamin 2 to self-assemble into rings is retained in dynamin 2-K562E. We next examined the direct effects of Bis-T-23 on dynamin polymerization. In the presence of Bis-T-23, polymerization of recombinant dynamin 2-WT increased by thirty times in comparison to that without Bis-T-23. On the other hand, polymerization of dynamin 2-K562E slightly increased by three and half times (Supplementary Figures S2E, F). These results suggested that self-assembly and membrane tubulating activity of dynamin 2-K562E was defective, and the differential effects of Bis-T-23 *in cellulo* on dynamin 2-WT and dynamin 2-K562E (Figure 3) could be attributed to changes in the molecular characteristics of dynamin 2-K562E.

**FIGURE 4:**
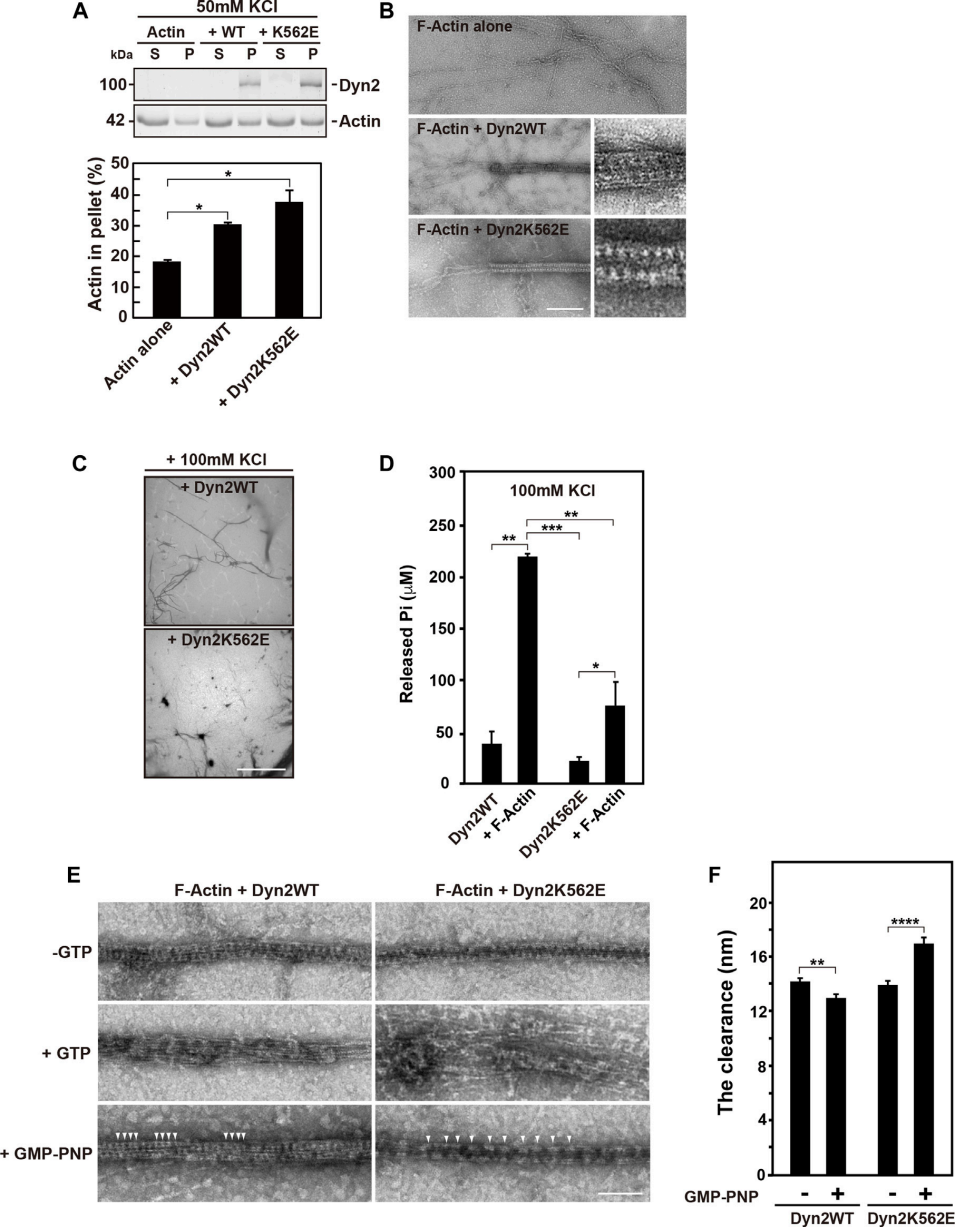
*In vitro* actin bundle formation by dynamin 2 (Dyn2)-WT or K562E. **(A)** Low-speed sedimentation assay for dynamin 2-WT- or K562E-induced actin bundle formation in low ionic strength buffer. The top panel shows actin in the pellet (P) or supernatant (S) fraction in the presence or absence of dynamin. Quantification of actin in the low-speed pellet in actin only, dynamin 2-WT, and K562E samples by densitometry (bottom panel). Data are means ± S.E.M. of three independent experiments. (*; *p* < 0.05). **(B)** Transmission electron micrographs of negatively-stained actin bundles formed by dynamin 2-WT (middle) or K562E (bottom). Scale bar: 200 nm, 50 nm in enlarged images. **(C)** Low magnification electron micrographs of negatively-stained actin bundles show a decrease in actin bundle formation by dynamin 2-K562E in the high ionic strength buffer. Scale bar: 8.5 μm. **(D)** Actin filaments (F-Actin) increases Pi release by dynamin 2-WT or K562E. Data are means ± S.E.M. of three independent experiments. (***; *p* < 0.001: **; *p* < 0.01: *; *p* < 0.05). **(E)** Electron micrographs showing morphological changes of dynamin 2-WT- or K562E-induced actin bundles in high ionic strength buffer. A reaction consisting of recombinant dynamin (1.5 μM) and F-Actin (1 μM) was initiated as in D, and the resulting actin bundles were treated with buffer alone, 0.1 mM GTP, or 0.5 mM GMP-PNP at room temperature for 5 min. Dynamin polymers were shown by arrowheads. Scale bar: 100 nm. **(F)** The clearance among adjacent dynamin 2-WT spiral polymer in the actin bundles. Twenty four (dynamin 2-WT/-GTP) or 27 (dynamin 2-WT/GMP-PNP), 21 (dynamin 2-K562E/-GTP) or 35 (dynamin 2-K562E/GMP-PNP) negatively-stained TEM images taken at ×30000 magnification from three independent experiments were used for the quantification. Data are means ± S.E.M. of three independent experiments. (****; *p* < 0.0001: **; *p* < 0.01).

Given that dynamin directly bundles actin filaments (Zhang et al., 2020), we assessed whether dynamin 2-WT or K562E bundles actin filaments. Preformed actin filaments were incubated *in vitro* with or without recombinant dynamin 2-WT or K562E, and actin bundle formation was examined by low-speed sedimentation assays. In the absence of dynamin 2 proteins, actin in the pellet after centrifugation at 14,000 x g was around 20% of total actin (Figure 4A). In the presence of dynamin 2-WT or dynamin 2-K562E, actin in the pellets increased by 30–40% (Figure 4A). These results suggested that dynamin 2-WT and K562E bundle actin filaments to similar extents.

TEM revealed that actin filaments were often tightly bundled by dynamin 2-WT or K562E (Figure 4B). The diameter of actin filament bundles in the presence of dynamin 2-WT and dynamin 2-K562E was 41.0 ± 0.6 nm (*n* = 24) and 41.4 ± 0.5 nm (*n* = 21), respectively. Periodically arranged dynamins were often observed both in dynamin 2-WT- and dynamin 2-K562E-induced actin bundles (Figure 4B inset). Atomic force microscopy (AFM) was used to investigate dynamin and actin filament configurations. AFM revealed that dynamin 2-WT ring-like structures formed spirals, and actin filaments directly bound to the outer rim of the dynamin spirals (Supplementary Figure S3). Dynamin 2-K562E bundled actin filaments in the same manner as that of dynamin 2-WT (Supplementary Figure S3).

Next, the effect of actin bundles on dynamin GTPase activity was determined. High ion strength buffer conditions containing 100 mM KCl were used to detect actin bundling-dependent GTPase activity because low ionic strength buffer containing 50 mM KCl causes a dynamin 2 self-assembly-dependent increase in GTPase activity (Warnock et al., 1997). Low magnification TEM images showed that dynamin 2-WT bundled actin filaments in the high ion strength buffer. By contrast, actin bundle formation by dynamin 2-K562E was lower than that of dynamin 2-WT (Figure 4C). Under the same conditions, concentration of released free phosphate by dynamin 2-WT or dynamin 2-K562E increased 6-fold or 4-fold, respectively, relative to that without actin proteins (Figure 4D).

We next examined morphological changes of actin bundles upon GTP hydrolysis of dynamins. The addition of GTP caused the rapid depolymerization of dynamin spiral polymers, and the resulting dispersion of actin bundles (Figure 4E middle panels) consistent with recent report (Zhang et al., 2020). This morphological change is likely to be largely dependent on GTP hydrolysis, although the reaction mixture also contains ATP for the purpose of stabilizing actin filaments. It is known that dynamin has much higher affinity to GTP compared to that to ATP (Maeda et al., 1992). Consistently, in the presence of nonhydrolyzable GTP analogue, GMP-PNP, did not result in disassembly of dynamin spiral polymers on the actin bundles (Figure 4E bottom panels). The clearance among adjacent dynamin 2-WT polymers in the actin bundles changed from 14.2 ± 0.2 nm (-GTP, n = 24) to 12.9 ± 0.3 nm (+GMP-PNP, *n* = 27). On the other hand, clearance among adjacent dynamin 2-K562E polymer in the actin bundles changed from 13.9 ± 0.3 nm (-GTP, *n* = 21) to 16.9 ± 0.5 nm (+GMP-PNP, *n* = 35) (Figures 4E bottom panels, F).

### Dynamin 2 Crosslinks Actin Bundles and Membranes, and Dynamin 2-K562E Reduces the Association Between Membranes and Actin Bundles

**FIGURE 5:**
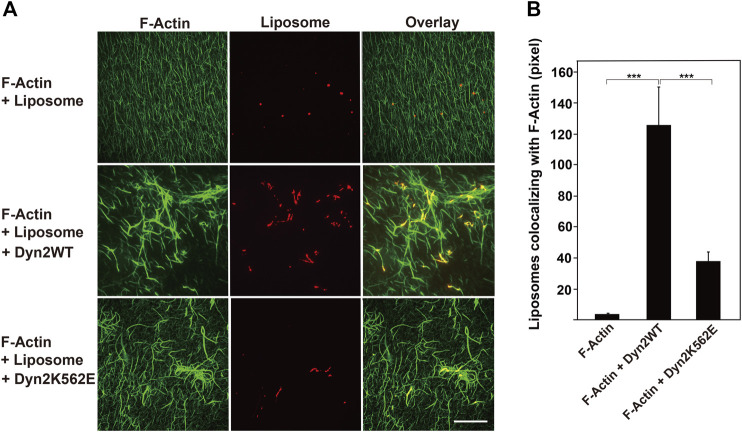
Decreased interaction of actin bundles formed by dynamin 2-K562E with PIP_2_-containing lipid vesicles. **(A)** Actin filaments (F-Actin) and lipid vesicles were visualized with Alexa Fluor 488-phalloidin (green) and Rhodamine-phosphatidylethanolamine (PE) (red), respectively. Preformed actin bundles by dynamin 2-WT (middle panels) or dynamin 2-K562E (bottom panels) were mixed with rhodamine-labeled lipid vesicles. Note that thick actin bundles formed by dynamin 2-WT associated with several lipid membranes. In the presence of dynamin 2-K562E, actin bundles were thinner and bound to few lipid vesicles. Scale bar: 20 μm. **(B)** Quantification of liposomes colocalizing with actin filaments. Data are means ± S.E.M. of 10 images (F-Actin), 25 images (F-Actin + Dyn2 WT) or 25 images (F-Actin + Dyn2 K562E) from three independent experiments. (***p < 0.001).

Dynamin binds to membrane phospho-lipids, such as PIP_2_ via its PH domain, and this association is essential for membrane deformation (Antonny et al., 2016). We therefore asked whether dynamin 2-induced actin bundles could bind to lipid membranes. Actin filaments did not form bundles or associate with lipid vesicles in the absence of dynamins (Figure 5A). In the presence of dynamin 2-WT, almost all actin filaments incorporated into thick actin bundles that colocalized with liposomes (Figure 5A). On the other hand, fewer actin bundles formed in the presence of dynamin 2-K562E than that in dynamin 2-WT (Figure 5A). Liposomes colocalizing with actin filaments or bundles in the presence of dynamin 2-K562E was approximately 30% of that in the presence of dynamin 2-WT (Figure 5B). These results indicate that dynamin 2 has actin-bundling and lipid-binding properties, and that dynamin 2-K562E has lower lipid-binding activity during actin bundling than that of dynamin 2-WT.

## Discussion

Two dynamin isoforms, dynamin 1 and dynamin 2, are expressed in podocytes (Soda et al., 2012). Dynamin 2 in podocytes play a role in maintaining the glomerular slit diaphragms by directly regulating actin (Gu et al., 2010; Schiffer et al., 2015) or by modulating endocytosis (Soda et al., 2012). However, the mode of action of actin regulation by dynamin 2 in podocytes remains unsolved. We have investigated the actin regulation by using variety of dynamin 2 CMT mutants to clarify CMT pathogenesis. Among the mutants, we found that expression of K562E, a member of CMT mutant in dynamin 2, resulted in the decrease of stress fibers and formation of actin clusters (Yamada et al., 2016b). We also examined two kinds of CMT mutants, G358R and 555Δ3. These CMT mutations did not affect the actin cytoskeleton (Yamada et al., 2016b). In the present study, we used the K562E mutants to clarify the function of dynamin 2 on actin cytoskeleton by the comparison of dynamin 2-WT and K562E in conditionally immortalized HPCs. Because the CMT mutations in dynamin 2 cause autosomal dominantly inherited diseases (Züchner et al., 2005), the expression of exogenous CMT mutant in cells would represent the pathological phenotype even though the presence of endogenous WT dynamin. In the study, we exogenously expressed dynamin CMT mutant in the presence of endogenous dynamin 2 to access the effect of actin filaments. Dynamin 2-K562E-expressing HPCs had lower stress fibers and actin filaments than those of dynamin 2-WT-expressing HPCs (Figures 1, 2). In addition, dynamin 2-K562E colocalized with aberrant actin clusters and bundles in areas, in which α-actinin-4 was located (Figure 1 and Supplementary Figure S1). Stress fiber reformation was lower in dynamin 2-K562E-expressing HPCs than that in dynamin 2-WT-expressing cells (Figure 2). These results suggest that dynamin 2 is involved in stress fiber formation in HPCs.

Bis-T-23, a dynamin polymerization enhancer (Schiffer et al., 2015), stimulated the formation of stress fibers and focal adhesions in dynamin 2-WT-expressing cells (Figure 3). On the other hand, Bis-T-23 had less effects on stress fiber formation and focal adhesion in dynamin 2-K562E-expressing cells than that in dynamin 2-WT-expressing cells (Figure 3). Moreover, Bis-T-23 was unable to stimulate dynamin 2-K562E polymerization *in vitro* (Supplementary Figure S2E). *In vitro* studies revealed lower dynamin 2-K562E self-assembly and membrane tubulation with decreased GTPase activity than that in dynamin 2-WT (Supplementary Figure S2). WT and mutant dynamins formed ring-like structures and/or spirals in the presence of actin filaments (Figure 4), and bound to actin filaments outside of the dynamin rings (Figure 4 and Supplementary Figure S3). Crosslinking between membranes and actin bundles triggered by dynamin 2-K562E were lower than those of dynamin 2-WT (Figure 5). These results indicate that proper self-assembly and association of dynamin 2 to membranes are crucial for actin regulation in HPCs.

Dynamin K562E has its mutation site in the PH domain at the interface of polymerized dynamin and membranes, and the mutant is defective in lipid-binding (Kenniston and Lemmon, 2010). In this study, dynamin 2-K562E hardly generated membrane tubules from liposomes, and therefore the mutant rarely polymerized into regularly arranged spirals on lipid membrane (Supplementary Figure S2C). The dynamin 2-K562E mutation in PH domain largely reduced membrane-binding ability, but not affected its actin binding capability (Figure 4A). Thus, the defective lipid-induced oligomerization of dynamin 2-K562E mutant might impact the proper formation of actin stress fibers in HPCs. Dynamin might have several putative actin binding sites. Gu and others determined several crucial amino acid residues for actin binding in the dynamin middle domain and upstream of the PH domain (Gu et al., 2010). Furthermore, the proline-rich domain is essential for actin bundling (Zhang et al., 2020). From the study, the K562E mutation seems to rarely affect its actin binding and bundling ability.

AFM revealed that several actin filaments bound to the outer rim of dynamin rings or spirals. Furthermore, actin filaments were not observed inside the dynamin spirals (Supplementary Figure S3). There are reports that the dynamin ring bundles actin filaments indirectly and directly (Yamada et al., 2013; Zhang et al., 2020). Dynamin 1 binds to cortactin, which is an actin filament binding protein (Wu and Parsons 1993), and forms a ring-shaped or spiral complex (Yamada et al., 2013). The dynamin-cortactin ring bundles actin filaments inside of the ring. The three-dimensional structure of the dynamin-actin filament complex needs to be determined at higher resolution such as by cryo-electron tomography reconstruction to better understand the nature of actin binding to dynamin.

Differentiated podocytes have a complex architecture with a multitude of foot processes that interdigitate with those of neighbouring podocytes to form and maintain the glomerular slit diaphragms (Pavenstädt et al., 2003). Actin serves as the main cytoskeletal structure in foot processes (Perico et al., 2016). Podocyte-specific conditional double knockouts of dynamins 1 and 2 in mice result in severe proteinuria and renal failure because of disruptions to the glomerular slit diaphragms (Soda et al., 2012). Dynamin 2 is thought to not only strengthen the actin cytoskeleton through its actin-bundling ability, but also promotes the formation of stress fibers and focal adhesions to maintain podocyte morphology and filtration functions. The present study shows that the self-assembly, and the membrane binding are essential characteristics of dynamin for the formation of stress fibers and actin bundles in HPCs.

Dynamin 2 binds to several actin-related proteins, and indirectly or directly regulates actin. Our previous studies report that another dynamin isoform, dynamin 1, is crucial for proper distribution and stability of microtubules in podocytes (La et al., 2020). Dynamin 2-dependent regulatory functions of stress fibers and focal adhesions in coordination with dynamin 1-dependent distribution and stabilization of microtubules, could be essential for normal podocyte function.

## Data Availability

The raw data supporting the conclusions of this article will be made available by the authors, without undue reservation.
