# Supplementary figures and images for: The Lipid-Binding Defective Dynamin 2 Mutant in Charcot-Marie-Tooth Disease Impairs Proper Actin Bundling and Actin Organization in Glomerular Podocytes
Source: Front Cell Dev Biol. 2022 May 10;10:884509. doi: 10.3389/fcell.2022.884509 (PMC9127447; doi:10.3389/fcell.2022.884509)

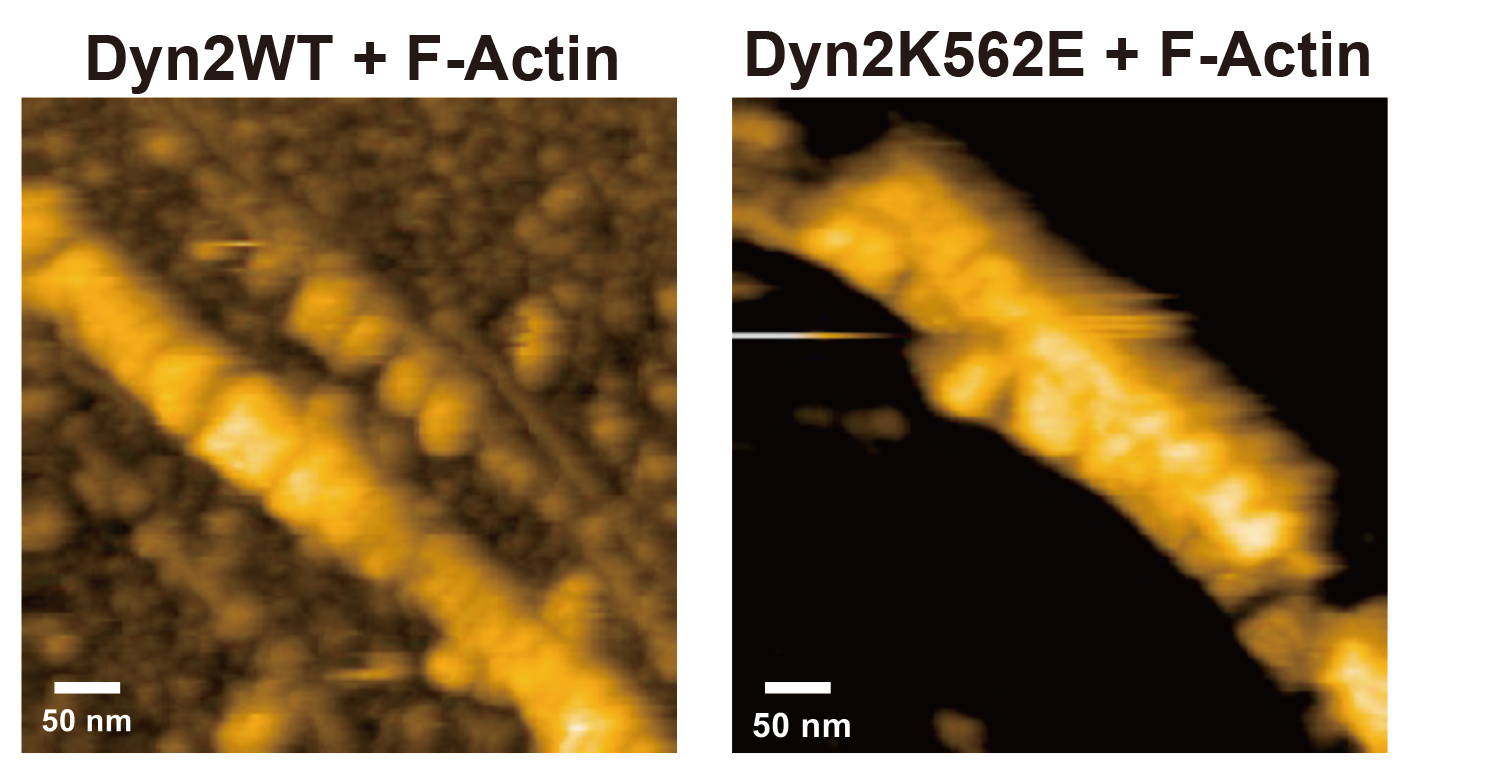

Supplement: Supplementary file 1 [file Image3.tif]

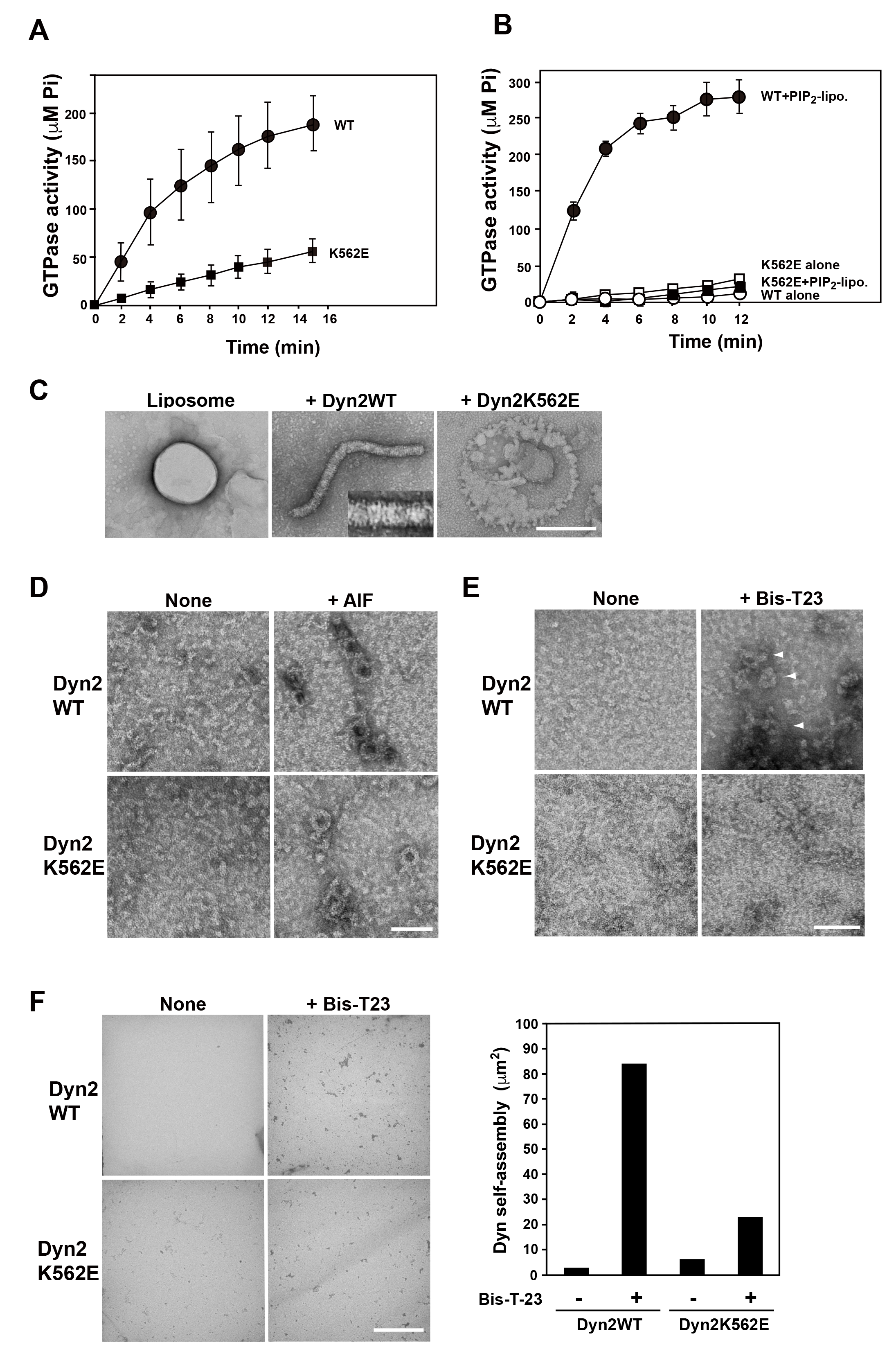

Supplement: Supplementary file 2 [file Image2.tif]

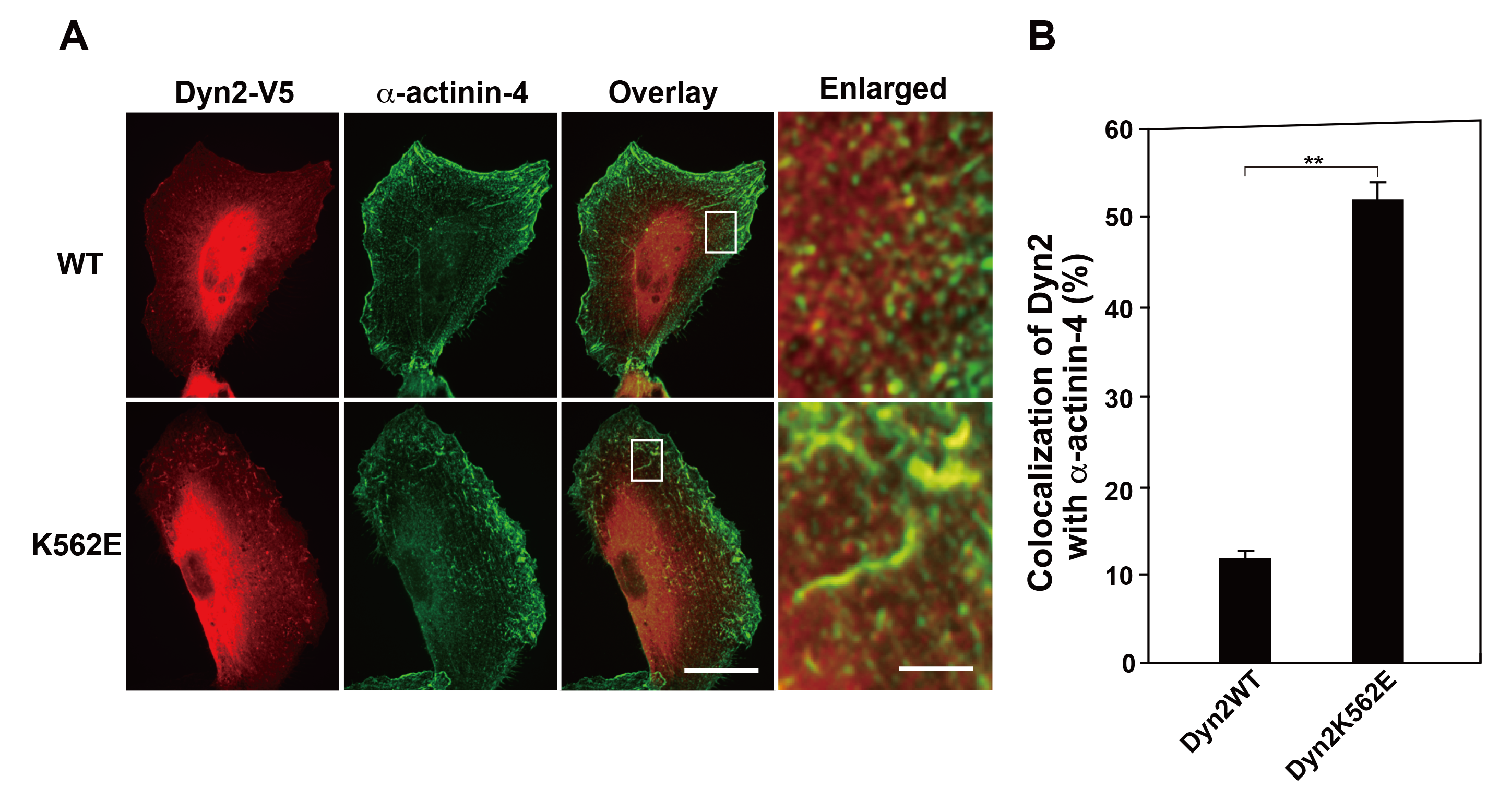

Supplement: Supplementary file 3 [file Image1.tif]
